# Predicting morbidity in older travellers during a short-term stay in the tropics: the ELDEST study

**DOI:** 10.1093/jtm/taaa216

**Published:** 2020-11-19

**Authors:** Jessica A Vlot, Marissa G D Vive, Henricus J Brockhoff, Pieter J J van Genderen, Marie-Christine E Trompenaars, James E van Steenbergen, Leonardus G Visser

**Affiliations:** Department of Infectious Diseases, Leiden University Medical Center, Albinusdreef 2, 2333 ZA Leiden, The Netherlands; Department of Infectious Diseases, Leiden University Medical Center, Albinusdreef 2, 2333 ZA Leiden, The Netherlands; Department of Infectious Diseases, Municipal Health Service, Westeinde 128, 2512 HE The Hague, The Netherlands; Harbour Hospital and Institute for Tropical Diseases, currently working on Department of Medical Microbiology and Infectious Diseases, Erasmus Medical Center, Doctor Molewaterplein 40, 3015 GD Rotterdam, The Netherlands; Department of Infectious Diseases, Municipal Health Service, Schiedamsedijk 95, 3011 EN Rotterdam, The Netherlands; Department of Infectious Diseases, Leiden University Medical Center, Albinusdreef 2, 2333 ZA Leiden, The Netherlands; Centre for Infectious Disease Control, Netherlands Institute for Public Health and Environment, Antonie van Leeuwenhoeklaan 9, 3721 MA Bilthoven, The Netherlands; Department of Infectious Diseases, Leiden University Medical Center, Albinusdreef 2, 2333 ZA Leiden, The Netherlands

**Keywords:** Older traveller, travel medicine, polypharmacy, comorbidity, grip strength, infectious diseases, predictors

## Abstract

**Background:**

Older persons may suffer more from travel-related health problems because of ageing and underlying chronic disorders. Knowledge on who is more likely to have these health problems helps to tailor travel health advice more specifically. This study aimed to determine predictors of travel-related morbidity in older travellers by assessing their pre-travel characteristics and performance using physical and cognitive functioning tests.

**Methods:**

Multicentre prospective cohort study among older travellers (≥60 years) who consulted one of the participating Dutch travel clinics. Handgrip strength and cognitive performance were measured pre-travel. Participants completed questionnaires before departure and 1 and 4 weeks after return. A diary recorded health complaints during travel until 2-week post-travel.

**Results:**

In total, 477 travellers completed the study (follow-up rate of 97%). Participants’ median age was 66 years. The most visited regions were South-East Asia (34%) and South Asia (14%). Median travel duration was 19 days. Polypharmacy (≥5 medications per day) was not uncommon (16%). The median Charlson Comorbidity Index (CCI) score was 0. Self-reported travel-related infectious diseases concerned primarily respiratory tract infections (21%) and gastroenteritis (10%) whereas non-infectious complaints were injuries (13%), peripheral edema (12%) and dehydration (3%). Medical assistance was sought by 18%, mostly post-travel from their general practitioner (87%). Self-reported physical and mental health-related quality of life significantly improved during and after travel. Predictors for an increased risk of travel-related morbidity were higher CCI score, more travel experience, longer travel duration, higher number of daily medications, visiting northern Africa or South-East and East Asia, and phone and social media use.

**Conclusion:**

Older Dutch travellers are generally fit, well-prepared and suffer not only from common infectious health problems, but also from injuries. Travel improved their self-perceived health. The predictors could be used to identify the more at-risk older traveller and to decrease travel-related morbidity by optimizing pre-travel advice.

## Introduction

Over the past decades, increase in life expectancy and vitality has led to a growing older population traveling internationally.^1^ In previous studies 15–30% of all international travellers were older adults.^2–4^ Between 1995 and 2017, the percentage of Dutch travellers to tropical destinations has almost doubled from 8% to 16%.^5^ It is conceivable that this also holds true for older travellers.

The travel-related morbidity of older travellers is expected to differ from that of younger travellers due to physiological, medical and behavioural differences.^6–9^ Due to their altered homeostasis older persons may suffer more from exposure to extreme climate and environmental conditions, potentially resulting in increased susceptibility to dehydration and hyper- or hypothermia.^10–13^ In addition, older persons are more susceptible to infections due to impaired immune responses, waning immunity and limited effectiveness of pre-travel vaccinations.^4^^,^^14–17^ Moreover, polypharmacy and underlying chronic disorders, including cardiovascular disease, diabetes mellitus (DM) and chronic respiratory diseases are more prevalent among older persons.^18–23^ This poses a risk of decreased physical functioning, drug-related side effects and drug–drug interactions or exacerbations of pre-existing illnesses during travel.^10^^,^^21^^,^^24–26^ Yet, older travellers choose different, possibly lower-risk destinations and travel modes and show less risky behaviour, which may diminish their travel-related health risks.^7^^,^^27^

A case-control study by Gautret et al. revealed that the observed proportion of illnesses, such as lower respiratory tract infections (RTIs), trauma and injuries, urinary tract infections and heart disease, was higher in the older travellers visiting a travel clinic post-travel compared with younger travellers.^6^ Illnesses such as acute diarrhoea, upper RTIs, mild malaria and dengue, were less frequently observed. However, the generalizability of these findings may be limited due to confounding by indication as only ill older travellers presenting themselves at the clinic post-travel were included.

Our aim was to identify predictors related to the occurrence of morbidity in older travellers during their tropical travel and shortly thereafter. To that end we evaluated their pre-travel performance using physical and cognitive functioning tests and determined the incidence, duration and inconvenience of travel-related morbidity in a prospective cohort.

## Methods

### Design and participants

We conducted a multicentre prospective observational cohort study among older adults traveling to tropical destinations (ELDEST study, morbidity in ELDErly travellers during a Short-Term stay abroad). Travellers were informed and recruited during their regular visit at four Dutch travel clinics between July 2016 and November 2017 [LUMC in Leiden (coordinating centre), Harbor Hospital Rotterdam and Municipal Health Services (MHSs) Rotterdam-Rijnmond and Haaglanden]. Inclusion criteria were: age ≥ 60 years, a scheduled travel to a tropical destination and a travel duration of ≤35 days. Exclusion criteria were inability to complete diary and questionnaires because of foreign language, or cognitive disability (i.e. suffering from memory disorders) or visiting the clinic less than 2 weeks prior to departure.

The study consisted of two parts. Part A collected pre-travel basic demographic information, and travellers completed physical and cognitive functioning tests. For logistical reasons, the cognitive test could not be performed at the MHSs sites. In part B, travellers completed pre- and post-travel questionnaires. In addition, a diary on travel-related health complaints was kept starting 1-week pre-travel, during travel until 2-week post-travel. Depending on their willingness to participate, travellers were included solely in part A or in both parts. This study was approved by the Medical Ethical Committee of the LUMC (registry number P16.056). All participants signed an informed consent. We followed the strengthening the reporting of observational studies in epidemiology (STROBE) reporting guideline.

### Functional tests (part A)

Hand grip strength is correlated with physical functioning and several important health outcomes.^28^ Grip strength was defined as the maximum strength from three attempts, measured with the Jamar Hydraulic hand dynamometer.^29^^,^^30^ The six item cognitive impairment test (6CIT) was conducted to assess the level of cognitive deficits.^31^^,^^32^ This test can be completed within 3–4 min and consists of six weighted items including one memory, two attention and three orientation questions. The 6CIT is not influenced by education level.^32^ A higher score is associated with more cognitive impairment (Supplementary Appendix 1).

### Questionnaires (part B)

Questionnaires were pre-tested among older adults for clarity and comprehensibility before start of the study. Travellers completed questionnaires at different time points: 1-week pre-travel, 1-week post-travel and 4-week post-travel. If travellers reported health complaints in the third questionnaire, an additional questionnaire was filled out 8-week post-travel.

Questionnaire 1 captured demographic data, medical history, medication and travel characteristics. In addition, three standardized tests were included to identify potential risk profiles based on health status, independence and (co)morbidity. Self-reported health status was assessed by the Short Form 36 health survey (SF-36) measuring eight health domains: physical functioning, social functioning, role limitations due to physical or emotional problems, mental health, vitality, bodily pain and general health perception.^33^ A higher SF-36 score is associated with a better health status (range 0–100). The level of independence of performing daily activities (e.g. dressing) was measured using the Katz activities of daily living (Katz-ADL).^34^^,^^35^ A higher Katz-ADL score is associated with more dependence (range 0–26). The comorbidity burden was assessed with the Charlson Comorbidity Index (CCI), a tool to measure comorbidity and to estimate 10-year survival (Supplementary Appendix 2).^36^^,^^37^ Questionnaire 2 concerned travel preparation, risk behaviour, health complaints and treatment. Post-travel health complaints and (possible) medical treatment were evaluated in questionnaire 3 and 4. The SF-36 was repeated twice to measure changes in self-reported health.

### Diary (part B)

Health complaints and experienced inconvenience were reported daily in a paper diary, starting 1-week pre-travel until 2-week post-travel. Every traveller received a digital thermometer for measuring body temperature in case of illness.

### Definitions

Polypharmacy was defined as the use of five or more medications per day (not including malaria prophylaxis).^38^ Diarrhoea was defined as the passage of three or more unformed stools during a 24-h period (WHO definition).^39^ Fever was defined as body temperature ≥ 38°C. Travel destinations were categorized according to geographical regions of the United Nations Statistics Division.^40^ Travel-related morbidity was categorized by evaluating the presence, duration, inconvenience and treatment of predefined symptom clusters using the diaries (Supplementary Appendix 3). Symptom clusters were defined on the presence of health complaints, matching the Dutch General Practitioners guidelines as closely as possible.^41^

## Sample size

We estimated that ~20% of older travellers would experience some kind of health problem during their foreign stay, but data for Dutch older travellers are lacking. Therefore, a formal power calculation was not performed. As many travellers as feasible were included within the timeframe of the project with the intention to collect complete data of at least 100 travellers aged between 70 and 79 years old, which would result in at least 20 travellers with health problems in this age group. Based on the age distribution of the participating centres in the past years and an attrition rate of 25%, a total of 625 inclusions were estimated to be required for this study to achieve this goal.

### Statistical analysis

Statistical analyses were conducted using SPSS software, version 23 (IBM Corp). Firstly, descriptive analyses and univariable analysis were used for demographical, (physical) health status and travel characteristics of travellers participating in A and B. Travellers aged 60–69 years were compared with travellers 70 years or older in our cohort regarding the pre-travel health characteristics. Secondly, incidence, duration, experienced inconvenience and treatment of travel-related morbidity were determined. SF-36 scores were compared using Wilcoxon signed rank test. Thirdly, logistic regression analyses were performed to identify predictors for travel-related morbidity using univariable and multivariable analysis. Without preselection from the univariable analysis, variables were entered in a multivariable logistic regression using backward elimination until the Akaike information criterion (*P* < 0.157) was minimized. In accordance with the guidelines for establishing prediction models, we selected the Akaike information criterion above the classic method of statistical significance with a *P*-value <  0.05 because otherwise important variables could be indicated as ‘non predictive’ due to the relatively small sample size.^42^ The Nagelkerke R,^2^ Hosmer and Lemeshow test, Brier score and c-index of the model were assessed to determine the performance of the model. The 6CIT total score could not be included in the model, as it was unavailable for travellers which were included at the MHSs. Associations were reported as odds ratios, 95% confidence intervals (CI) and *P*-values.

## Results

### Study population and travel characteristics

**Figure 1:**
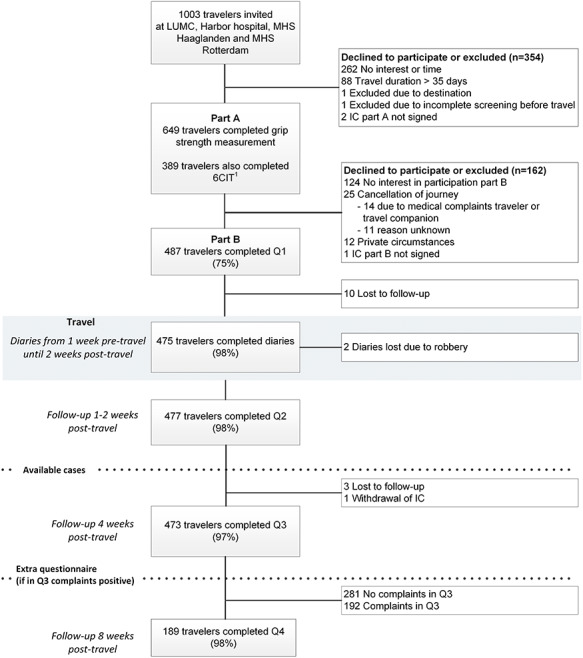
Flowchart of participants in the ELDEST study. ^1^ Only performed by participating travelers at two of the four clinics. 6CIT, Six Item Cognitive Impairment Test; IC, Informed Consent; LUMC, Leiden University Medical Centre; MHS, Municipal Health Service; Q, Questionnaire.

In total, 1003 travellers were invited, of whom 649 were included in part A (35% non-participation). Of these, 477 travellers (73%) were available for case analysis (Part A and B, follow-up rate questionnaires 97%) (Figure 1). Demographic and travel characteristics are shown in Supplementary Table S1. The median age was 66 years [interquartile range (IQR) 63–70]; 132 (28%) were aged 70 years and over.

Travellers were generally fit with an overall median 6CIT total score of 0 (IQR 0–2) and a median grip strength of 34 kg (IQR 28–45) (Table 1). The most visited regions were South-Eastern Asia (34%), and Southern Asia (14%) (Supplementary Figure S1). The median time spent abroad was 19 days (IQR 14–23). Almost all travellers owned a mobile phone (98%) and many used social media (60%) (Supplementary Table S1).

**Table 1:** Pre-travel health characteristics of 477 older travellers to the tropics

	**Available Cases All ages** ^**a**^ ***N* = 477**	**Available Cases 60–69 years** ***N* = 345**	**Available Cases ≥ 70 years** ***N* = 132**	**Comparison** *P***-value**^**h**^
**BMI,** *kg/m^2^, median (IQR)*	25.4 (23–28)	25.4 (23–28)	24.9 (23–28)	*P* = 0.514^i^
**Sensory function**				
Wearing glasses or contact lenses^b^	366 (77)	261 (77)	105 (80)	*P* = 0.395
Wearing hearing aid^b^	53 (11)	25 (7)	28 (21)	*P* < 0.001
**Influenza vaccination received in the past year** ^b^	265 (56)	167 (48)	98 (74)	*P* < 0.001
**Katz-ADL score**, *median (IQR)*^bc^	0 (0–0)	0 (0–0)	0 (0–0)	*P* = 0.138
0	461 (97)	336 (98)	125 (95)	
1	15 (3)	8 (2)	7 (5)	
**Grip strength,** *kg, median (IQR)*^d^	34 (28–45)	35 (28–46)	32 (25–42)	*P* < 0.001^i^
**6CIT total score** *, median (IQR)* ^e^	*N = 303* 0 (0–2)	*N = 225* 0 (0–2)	*N = 78* 2 (0–4)	*P* = 0.017
**CCI score** *, median (IQR)*	0 (0–1)	0 (0–1)	1 (0–2)	*P* < 0.001
0	287 (60)	226 (66)	61 (46)	
1	79 (17)	58 (17)	21 (16)	
2	66 (14)	37 (11)	29 (22)	
3	23 (5)	13 (4)	10 (8)	
4	8 (2)	3 (1)	5 (4)	
≥5	14 (3)	8 (2)	6 (4)	
**Number of medication per day**, *median (IQR)*	1 (0–3)	1 (0–3)	1 (0–4)	*P* = 0.011
None	159 (33)	125 (36)	34 (26)	
1–5	271 (57)	195 (57)	76 (58)	
6–10	42 (9)	23 (7)	19 (14)	
11–13	5 (1)	2 (1)	3 (2)	
**Polypharmacy** (≥5 medications per day)	77 (16)	45 (13)	32 (24)	*P* = 0.003
**Medical history**				
Any cardiovascular disease^f^	212 (44)	131 (38)	81 (61)	–
Hypertension	134	88 (26)	46 (36)	*P* = 0.039
Cardiac arrhythmia	32	16 (5)	16 (12)	*P* = 0.003
Myocardial infarct	13	7	6	*P* = 0.204
Angina pectoris	5	3	2	*P* = 0.620
Cardiac failure	3	2	1	–
Malignancy	76 (16)	41 (12)	35 (27)	*P* < 0.001
With metastases	8	5	3	
Skin disease	49 (10)	34	15	*P* = 0.627
Urinary tract infection(s) < 12-month pre-travel^b^	36 (8)	26	10	*P* = 0.995
Pulmonary disease	39 (8)	29	10	*P* = 0.767
Asthma	22	19	3	
COPD	14	9	5	
Auto-immune disorder^b^	32 (7)	18	14	*P* = 0.036
Diabetes mellitus	30 (6)	19 (6)	11 (8)	*P* = 0.259
With complications	7	5	2	
Gastric disease	22 (5)	17	5	*P* = 0.808
Intestinal disease	16 (3)	10	6	*P* = 0.397
Ulcerative colitis/Crohn’s disease	3	2	1	
Renal disease	15 (3)	8	7	*P* = 0.138
Kidney transplant	5	3	2	
Liver disease	6 (1)	4	2	*P* = 0.671
HIV	1 (0)	0	1	–
Dementia^g^	1 (0)	0	1	–

Data represent absolute numbers (N) and percentages (%), unless otherwise specified. Abbreviations: BMI, body mass index; IQR, interquartile range; Katz-ADL, Katz activities of daily living; 6CIT, Six Item Cognitive Impairment Test;; CCI, Charlson comorbidity index; COPD, Chronic obstructive pulmonary disease; HIV, human immunodeficiency virus.

^a^Available cases are eligible travellers that participated in part A and B.

^b^Percentages were calculated over the total number of travellers that answered the concerning question. Some travellers did not fill in the questions concerning these items, resulting in a maximum of three missing values.

^c^Katz-ADL: range 0–26.

^d^Grip strength: range 0–90 kg. For procedure, see Supplementary Appendix 1.

^e^6CIT: range 0–28. For procedure, see Supplementary Appendix 1. Measurements were unavailable for travellers included at the Municipal Health Services.

^f^This group variable represents the number of cardiovascular events including hypertension (*n* = 134), cardiac arrhythmia (*n* = 32), myocardial infarct (*n* = 13), angina pectoris (*n* = 5), cardiac failure (*n* = 3), transient ischemic attack (*n* = 8), cerebral infarction (*n* = 3) peripheral vascular disease (*n* = 14). Some travellers had multiple cardiovascular events therefore no comparison could be calculated between both age groups.

^g^Early vascular dementia; researchers decided that participating partner was allowed to help filling in the questionnaires and diary.

^h^Pearson’s Chi-Square test or Fisher Exact test was used, unless otherwise specified.

^i^Unpaired *t*-test was used.

### Pre-travel health characteristics

Polypharmacy was not uncommon among participants. One third of all travellers did not use any medication at all; 16% used five or more daily medications. In travellers aged 70 years and over polypharmacy occurred more frequently (*P* = 0.003). Katz-ADL revealed that 97% of travellers could be classified as independent. The median CCI was 0 (IQR 0–1), corresponding with an estimated 10-year survival of 92%.^36^ Pre-travel performance scores, particularly grip strength, 6CIT and CCI, were significantly worse in travellers aged ≥ 70 years. Overall, the three most observed pre-existing conditions were cardiovascular diseases (44%, mainly hypertension), malignancies (16%) and skin diseases (10%) (Table 1).

### Travel preparation

The majority of travellers booked their travel online (60%). They frequently consulted sources for advice, such as the general practitioner (GP, 91%) and the internet (18%). Nearly all travellers had a travel insurance (97%). Travellers often carried self-medication for diarrhoea such as loperamide (73%), and oral rehydration solution (ORS, 60%) (Supplementary Table S2). Also hand-hygiene products, such as hand sanitizers (58%), were brought along of which 75% (207/275) used these regularly (Supplementary Table S3).

### Risk behaviour

Travellers showed various kinds of risk behaviour such as consuming unpeeled fruit (76%), raw food products (27%, i.e. crustaceans or shellfish) or eating at street vendors (19%). Regular hand washing before a meal was practiced by 84% of travellers. About 20% reported contact with animals that mostly involved monkeys (91%) or dogs (48%) (Supplementary Table S3).

### Malaria prophylaxis

If malaria prophylaxis was indicated (*n* = 147), chemoprophylaxis and mosquito nets were used in 82% and 80% of travellers, respectively. Atovaquone/proguanil was mostly used (93%); only one traveller used mefloquine (1%). Most travellers were fully compliant (92%). Side effects are listed in Supplementary Table S4. No cases of malaria were reported.

### Morbidity during and shortly after travel

One out of 5 travellers (21%) suffered from a RTI, of which 1 in 10 travellers had a pre-existent pulmonary disease [e.g. asthma or chronic obstructive pulmonary disease (COPD)]. Almost one third of the RTIs (27%) were complicated (Table 2). One in six travellers with an uncomplicated RTI also reported these complaints pre-travel. Among all 98 travellers with an RTI, 30% experienced inconvenience, 16% used an antibiotic and 12% consulted a doctor.

**Table 2:** Self-reported morbidity in 475 older travellers traveling during and up to 2 weeks after travel to the tropics^a^

	**Cumulative incidence**	**Incidence density (cases/100 travel months)**		**Mean duration of complaints in days (range)** ^**f**^		**Number of travellers with symptoms in the week before travel**	**Number of travellers forced to alter program or confined to accommodation**	**Medication used**				**Medical assistance** ^**g**^
								**Antibiotic**	**Loperamide**	**Activated carbon**	**ORS**	
**Symptom clusters** ^**b**^	N (%)	Travel months:	512	Mean	Range	N (%)	N (%)	N (%)	N (%)	N (%)	N (%)	N (%)
**Infections (general)** ^**c**^	21 (4)		4.1	2.4	(1–7)	0	12 (57)	3 (14)	0	0	0	4 (19)
**Gastroenteritis**												
Uncomplicated	41 (9)		8.0	1.8	(1–5)	3 (7)	19 (46)	3 (7)	16 (39)	3 (7)	13 (32)	8 (20)
Complicated	6 (1)		1.2	3.5	(1–7)	1 (17)	6 (100)	2 (33)	3 (50)	2 (33)	2 (33)	
**Dehydration** ^**d**^	18 (4)		3.5	4.9	(1–18)	1 (6)	8 (44)	0	0	0	2 (11)	1 (6)
**Respiratory tract infection**												
Uncomplicated	72 (15)		14.1	3.1	(1–7)	12 (17)	23 (32)	10 (14)	0	0	0	12 (17)
Complicated	26 (6)		5.1	12.2	(4–21)	0	6 (23)	6 (23)	0	0	0	
**Urinary tract infection**												
Uncomplicated	2 (1)		0.4	1.0	(1–1)	0	0	1 (50)	0	0	0	0
Complicated	2 (1)		0.4	28.0	(20–36)	1 (5)	0	1 (50)	0	0	0	
**Cardiovascular complaints**												
Angina pectoris	5 (1)		1.0	5.4	(1–21)	2 (40)	1 (20)	0	0	0	0	1 (20)
Cardiac failure	3 (1)		0.6	4.0	(2–6)	1 (33)	1 (33)	0	0	0	0	
**Peripheral edema**	59 (12)		11.5	9.8	(3–25)	2 (3)	9 (15)	0	0	0	0	2 (3)
**Musculoskeletal complaints** ^**e**^	11 (2)		2.1	6.6	(1–14)	0	5 (45)	0	0	0	0	2 (18)
**Total**	**266 (56)**		**52.0**			**23 (9)**	**90 (34)**	**26 (10)**	**19 (7)**	**5 (2)**	**17 (6)**	**30 (11)**

Data represent absolute numbers (N) and percentages (%), unless otherwise specified. Abbreviation: ORS oral rehydration solution.

^a^Two diaries were robbed, resulting in 475 diaries.

^b^Data represent the number of cases fulfilling criteria of defined symptom clusters, see Supplementary Appendix 3. Some travellers fulfilled the criteria for multiple symptom clusters.

^c^Infections that did not fulfil criteria of another symptom cluster.

^d^Dehydration was observed in 18 travellers, of whom six travellers also experienced gastroenteritis.

^e^Three travellers reported pre-travel ‘stiffness’ and one was known with rheumatoid arthritis.

^f^Duration of complaints meeting symptom cluster criteria.

^g^Represents medical assistance needed for same symptom cluster, as indicated in questionnaire or diary.

**Table 3:** Medical consultations due to possible travel-related illnesses during the travel and post-travel period

	**During travel *N* = 7**	**1–2 weeks post-travel *N* = 33**	**2–4 weeks post-travel *N* = 34** ^**a**^	**4–8 weeks post-travel *N* = 22** ^**a**^	**Subtotal post-travel *N* = 79**	**Total study period *N* = 84**
**Type of medical assistance** ^b^						
General practitioner	4 (57)	28 (85)^d^	27 (79)	14 (61)	69 (87)	73 (87)
Medical specialist	0	2 (6)	6 (18)^e^	7 (30)	15 (19)	15 (18)
Emergency room	3 (43)	0	0	0	0	3 (4)
Hospital admission	0	3 (9)^f^	1 (3)^g^	1 (4)^h^	5 (6)	5 (6)
**Reason for seeking medical assistance**						
Infections (general)	0	2 (6)	2 (6)	0	4 (5)	4 (5)
Gastrointestinal complaints	3 (43)	6 (18)	4 (12)	5 (22)	15 (19)	18 (21)
Dehydration	0	1 (3)	0	0	1 (1)	1 (1)
Respiratory complaints	0	7 (21)	10 (29)	6 (26)	23 (29)	23 (27)
Urinary tract complaints	0	1 (3)	2 (6)	3 (13)	6 (8)	6 (7)
Cardiovascular complaints	0	1 (3)	3 (9)	0	4 (5)	4 (5)
Peripheral edema	0	3 (9)	0	0	3 (4)	3 (4)
Musculoskeletal complaints	0	3 (9)	6 (18)	3 (13)	12 (15)	12 (14)
Ear nose throat complaints	1 (14)	6 (18)	2 (6)	3 (13)	11 (14)	12 (14)
Other	3 (43)	3 (9)	5 (15)	2 (9)	10 (13)	13 (15)
**Total medical consultations** ^**c**^	7 (100)	33 (100)	34 (100)	22 (96)	89 (113)	97 (115)

Data represent absolute numbers (N) and percentages (%). Medical support for complaints that were (possibly) travel-related, as was judged by a clinician.

Regular medical appointments such as annual influenza vaccination of measuring blood pressure were excluded.

^a^In the period of 2–4 weeks post-travel, three travellers were lost to follow-up. In the period 4–8 weeks post-travel, an additional three travellers who did reported medical complaints in questionnaire 3 did not fill in questionnaire 4.

^b^Travellers that received multiple types of medical assistance were only indicated once in the table at the highest level of care that was received.

^c^Totals may exceed 100% since some travellers sought medical support multiple times (10 travellers twice, 1 traveller thrice).

^d^Among which one traveller with African tick bite fever.

^e^Among which one traveller with scabies.

^f^Dehydration due to gastroenteritis, acute cardiac failure and arthritis.

^g^Sepsis.

^h^Acute cholecystitis.

**Table 4:** Best predicting characteristics for developing travel-related morbidity in 475 older travellers to the tropics

	**No morbidity**	**Morbidity**	**Univariable analysis**		**Multivariable analysis**	
	***N* = 281**	***N* = 194**	**OR [95% CI]**	*P* **value**	**OR [95% CI]**	*P* **value**
**Gender, female**	131 (47)	100 (52)	1.22 [0.84–1.78]	0.29		
**Age** *, years, median (IQR)*	66 (62–70)	66 (63–71)	1.02 [0.98–1.05]	0.30		
**Educational level**						
Primary education (=ref)	19 (7)	19 (10)	1.00	0.04	1.00	0.03
Secondary education	85 (30)	75 (39)	0.88 [0.44–1.79]		0.80 [0.37–1.74]	
Higher education	177 (63)	100 (52)	0.57 [0.27–1.12]		0.48 [0.23–1.02]	
**Immigrant**	23 (8)	15 (8%)	0.94 [0.48–1.85]	0.86		
**Travel advice obtained at MHS**	101 (36)	71 (37)	1.03 [0.70–1.50]	0.88		
**Travel experience to tropics: number of journeys in preceding 5 years,** *median (IQR)*	2 (1–5)	2 (1–5)	1.00 [0.98–1.02]	0.95	1.05 [0.985–1.13]	0.13
**Tropical travel destination**						
Caribbean, Central and South America (=ref)	44 (16)	36 (19)	1.00	0.03	1.00	0.02
Northern Africa	4 (1)	7 (4)	2.14 [0.58–7.88]		3.8 [0.91–15.82]	
Sub Saharan Africa	88 (31)	37 (19)	0.51 [0.29–0.92]		0.63 [0.34–1.20]	
Central, South and Western Asia/Middle East	102 (36)	76 (39)	0.91 [0.54–1.55]		0.87 [0.49–1.56]	
South-East and East Asia	42 (15)	38 (20)	1.11 [0.59–2.06]		1.56 [0.78–3.11]	
**Travel duration,** *days, median (IQR)*	18 (14–23)	20 (15–24)	1.03 [1.01–1.06]	0.02	1.04 [1.004–1.070]	0.03
**Duration between pre-travel consult and departure,** *days, median (IQR)*	38 (21–52)	37 (22–50)	1.00 [0.99–1.00]	0.44		
**Purpose of travel**						
Business (=ref)	10 (4)	5 (3)	1.00	0.79		
Visiting friends or relatives	40 (14)	30 (15)	1.50 [0.46–4.85]			
All other	231 (82)	159 (82)	1.38 [0.46–4.10]			
**Travel group composition**						
With an organized group travel (=ref)	50 (18)	46 (24)	1.00	0.17	1.00	0.12
With family	191 (68)	116 (60)	0.66 [0.42–1.05]		0.63 [0.38–1.04]	
All other	40 (14)	32 (16)	0.87 [0.47–1.61]		0.94 [0.47–1.85]	
**Type of accommodation during travel**						
Accommodation owned by participant/family/friends (=ref)	26 (9)	20 (10)	1.00	0.43		
Luxurious rented accomodation	241 (86)	159 (82)	0.86 [0.47–1.60]			
Nonluxurious accomodation	13 (5)	15 (8)	1.39 [0.55–3.54]			
**Phone use**						
No phone (=ref)	7 (2)	4 (2)	1.00	0.16	1.00	0.11
Regular mobile phone	33 (12)	35 (18)	1.86 [0.50–6.93]		2.24 [0.53–9.44]	
Smartphone	241 (86)	155 (80)	1.13 [0.32–3.91]		1.21 [0.31–4.77]	
**Social media use**	166 (59)	119 (61)	1.08 [0.74–1.57]	0.69	1.55 [0.996–2.42]	0.05
**BMI, *kg/m*** ^***2,***^ *median, (IQR)*	25 (23–27)	25 (23–28)	1.02 [0.97–1.07]	0.51		
**Sensory function**						
Wearing hearing aid	30 (11)	23 (12)	1.13 [0.63–2.01]	0.67		
Wearing glasses or contact lenses	220 (78)	144 (74)	0.82 [0.53–1.25]	0.35		
**Katz-ADL score**, *median (IQR)*	0 (0–0)	0 (0–0)	1.23 [0.46–3.60]	0.63		
**6CIT total score** *, median (IQR)* ^a^	0 (0–2)	2 (2–2)	1.13 [1.01–1.27]	0.04		
**Grip strength,** *kg, median (IQR)*	36 (28–46)	32 (28–44)	0.99 [0.97–1.01]	0.15		
**Influenza vaccination received < 1 year**	147 (53)	116 (60)	1.13 [0.91–1.92]	0.14		
**Number of daily medications,** *median (IQR)*	1 (0–3)	2 (0–4)	1.13 [1.05–1.22]	0.001	1.07 [0.977–1.17]	0.14
**CCI score** *, median (IQR)*	0 (0–1)	0 (0–2)	1.20 [1.06–1.35]	0.003	1.15 [1.006–1.31]	0.04
**SF-36 sum scores pre-travel** *, median (IQR)*						
Physical health	92 (85–95)	87 (77–93)	0.97 [0.95–0.98]	<0.001		
Mental health	90 (86–94)	87 (79–91)	0.96 [0.94–0.98]	<0.001	0.96 [0.944–0.981]	<0.001

Data represent absolute numbers (N) and percentages per category (%), unless otherwise specified. Variables were selected for the multivariable regression analysis using backward selection until the Akaike information criterion (*P* ≤ 0.157) was satisfied. Abbreviations: OR, odds ratio; IQR, interquartile range; ref, reference; MHS, Municipal Health Service; BMI, body mass index; Katz-ADL, Katz activities of daily living; 6CIT, Six Item Cognitive Impairment Test; CCI, Charlson comorbidity index; SF-36, Short-Form 36 survey.

^a^Not included in the multivariable regression analyses since measurements were unavailable for travellers included at the Municipal Health Services. Model: constant = 9.02, Nagelkerke R^2^ = 0.18, Hosmer and Lemeshow test *P* = 0.53, c-index = 0.71, Brier score = 0.2087.

Gastroenteritis (GE) was observed in 47 travellers (10%), of which 6 (13%) were complicated (Table 2). One in five travellers had a gastrointestinal disease in their medical history, mostly gastroesophageal reflux or irritable bowel syndrome. Almost half of the travellers (46%) with uncomplicated and all complicated GE experienced inconvenience and altered their program or stayed in their accommodation. Eight travellers (20%) consulted a doctor and many used self-medication. The relative risk (RR) of contracting GE appeared to be higher for travellers using a proton-pump inhibitor, but was not significant (RR = 1.75, 95% CI 0.97 to 3.18, *P* = 0.07). Dehydration occurred in 18 travellers (4%), of whom 6 had GE, and 4 were taking diuretics. Two dehydrated travellers (11%) used ORS.

Despite the presence of comorbidities in 40% of travellers, exacerbations of pre-existent conditions were reported in only 5% of the travellers (Supplementary Table S5). Eight travellers (2%) experienced cardiovascular complaints; 88% had a pre-existing cardiovascular disease such as hypertension or cardiac arrhythmias (Table 2). One traveller with cardiac arrhythmia consulted a doctor. In 59 travellers (12%) with peripheral edema, 47% had a medical history of cardiovascular disease (mostly hypertension). A higher comorbidity burden (CCI) and the use of more daily medication was associated with a higher travel-related morbidity (*P* = 0.04 and *P* = 0.14 respectively, data not shown).

A total of 61 travellers (13%) suffered mostly minor injuries, such as cuts, abrasions or spraining (Supplementary Table S5) often caused by accidental falling (30%). No fractures were reported. One traveller reported a dog bite (WHO category II) in Malawi.

### Medical assistance

Medical assistance for health complaints possibly related to their travel was primarily sought between the first week to 2 months after return (81%), mostly at the GP (87%). Respiratory (27%) and gastrointestinal complaints (21%) were the main reasons of consultation. Only seven travellers consulted a GP (57%) or an emergency room (43%) during travel, mostly for gastrointestinal complaints, wounds or altitude sickness. Five travellers were hospitalized post-travel, none during travel. No mortality was observed (Table 3).

### Self-reported health

There was a significant improvement in mental health sum score and associated domains during travel (all *P* < 0.001). Improvement in self-perceived physical health was observed within the bodily pain domain (*P* < 0.001, data not shown). After travel, there were still significant improvements noticeable in the mental health sum score, vitality and general mental health (all *P* < 0.001) in comparison with pre-travel. In addition, a significant improvement in the physical health sum score (*P* = 0.01) and bodily pain (*P* < 0.001) were observed (Supplementary Table S6).

### Predictors for travel-related morbidity

Multivariable analysis demonstrated that traveling to Northern Africa or South-East and East Asia, phone and social media use, higher CCI score, higher number of medications per day, more tropical travel experience and longer travel duration seemed to be associated with increasing odds for travel-related morbidity. Travellers with a better SF-36 mental health sum score pre-travel, traveling with family and travellers with higher education appeared to have reduced odds for travel-related morbidity. Grip strength and Katz-ADL were not identified as predictors (Table 4).

## Discussion

In this multicentre prospective study we assessed whether physical and cognitive performance tests could predict travel-related morbidity in Dutch older travellers during a short-term stay in the tropics. We found that a higher CCI score and higher number of daily medications, but also more tropical experience, longer travel duration, traveling to Northern Africa or South-East and East Asia, and phone and social media use were associated with higher odds for travel-related morbidity. The participants were generally experienced and well-educated, physically and mentally fit with little (co)morbidity or polypharmacy and well-connected to the digital world of internet, social media and smartphones. As expected, travellers in higher age groups scored worse on performance measurements although these groups were not identified as predictors.

Our cohort had considerable travel experience, was well-prepared for their tropical trip, and showed limited risk-seeking behaviour. This last aspect is in line with the retrospective study of Alon et al. who demonstrated that older travellers practiced less risky eating and drinking habits and were more compliant with anti-malarial chemoprophylaxis than younger travellers.^7^ Most older travellers in our cohort were fully compliant (92%). It is noteworthy that a substantial number of our travellers used a smartphone and social media. This could imply that these methods of communication could be used for future (intervention) strategies as mobile technology will impact travel medicine more and more.^43–45^

In accordance with previous literature,^6^^,^^7^ older travellers frequently experienced ‘classic’ travel-related morbidities, such as gastrointestinal and respiratory infections, but they also reported complaints which are more likely to occur in older people such as dehydration (4%), cardiovascular complaints (2%), peripheral edema (12%) and accidental falls (4%). Unexpectedly, exacerbations of pre-existing illness were only rarely reported. In the post-travel questionnaire 141 travellers (30%) reported to have experienced diarrhoea, of whom 12 travellers did not temporarily stop their diuretics. Since older persons are more prone to complications such as hypotension or renal failure following dehydration, it is important to discuss during the pre-travel consult in which situations diuretics should be discontinued (e.g. during periods of vomiting and/or diarrhoea).^46^ Less anticipated complaints were injuries (13%), skin (12%) and musculoskeletal complaints (2%). Especially falling deserves further investigation, as falls are a major determinant of morbidity and mortality in older adults.^47^^,^^48^

Medical assistance was frequently sought, but mostly post-travel. Underlying reasons for late medical consultation were not studied. Possible explanations are previous experience with similar complaints that appear to be self-limiting, preference for own GP, or unexpected longer duration of complaints after travel.^49^

Our findings that older travellers experienced significant improvement in the self-reported mental- and physical health during and after travel extend earlier findings.^50–52^ This kind of effects of travel appears to have a positive effect on the perceived health of the traveller and could therefore outweigh the impact of health problems. Although this positive effect decreased on return, travellers still experienced improved health as compared with before travel.^50^ A survey study on the well-being and health among employees of German companies after a, mostly European, holiday revealed that enough leisure time, warmer destinations, being physically active, good sleep and making new social contacts facilitate improved health whereas dealing with a greater time-zone difference (i.e. jetlag) was associated with a decreased health.^52^ Most of our study participants travelled to warmer tropical destinations, whereby they frequently cross different time-zones. Interestingly, in the employee study an older subgroup aged 50–62 (18%) was analysed, and they found that age was associated with differences in holiday organization (e.g. duration and travel time), but did not affect the positive health changes on its own.^52^ The findings in our study cohort underline that traveling to the tropics does not only entail morbidity for the older traveller, but could positively affect both their mental and physical health. We did not address possible improvements on existing co-morbidities.

We hypothesized that pre-travel, validated physical and mental health performance measures might identify older travellers more at risk for travel-related morbidity. Grip strength was chosen as an objective measure of physical performance, but no association was found. The same holds for the level of independence measured with Katz-ADL. A possible explanation for these findings could be that most included travellers are fit and living an active and independent life, what enables them to undertake tropical journeys.

Finally, we identified several demographic (phone and social media use), travel (duration, destination, experience) and health characteristics (CCI and medication) as predictors of travel-related morbidity. We used the Akaike information criterion in order not to overlook any important associations. In line with our findings, previous research also identified travel duration and destination as predictors, but not specifically in the older traveller.^50^^,^^53^ Interestingly, age was not found to be an independent predictor for morbidity, even though pre-travel characteristics were found to be significantly different when comparing age groups (Table 1). The direction of some effect sizes seems counterintuitive (e.g. more travel experience seems to be associated with higher morbidity rates). It is therefore important to note that this model aimed to identify prognostic and not etiologic relationships between characteristics and travel-related morbidity. This relationship might be confounded by other characteristics, such as risk behaviour.^12^^,^^53^ Risk behaviour was not included in the multivariable regression model, since this cannot validly be measured pre-travel.^54^ The identified predictors could be used to identify older travellers with a relatively high risk for travel-related morbidity. Most of them could be easily assessed since they are part of the current pre-travel consultation (destination, duration and travel experience) or could easily be assessed at that moment (educational level, phone and social media use and CCI score). Future research should be conducted to confirm the association between the identified predictors and travel-related morbidity.

The strengths of this study are the multicentre approach, the large sample size of >100 travellers aged 70 and over, the high follow-up rate of 97%, the limitation of recall bias using a diary during travel and questionnaire shortly after return, the use of validated measurement tools, the use of well-defined symptom clusters to quantify reported morbidity and the collection of baseline data before departure which limits selection bias and offers the opportunity to compare health status pre- and post-travel.

The study also has some limitations that need to be discussed. This study population might not be completely representative for the older population in general (‘healthy traveller bias’). Compared with travellers who participated only in part A or partly in B, travellers who participated in A and B were slightly younger, had a higher grip strength and were less cognitively impaired (Supplementary Table S7). Also, when other aspects are taken into account, our travellers appeared to be fitter than the average age-matched Dutch population. A pre-existing illness was reported by 40% of our travellers, which is lower than the anticipated 50% in the general non-travelling Dutch population of 65 years and older,^55^ but comparable with the 43% in Swiss travellers who also visited non-tropical destionations.^56^ Also polypharmacy was somewhat lower in our study population (16%) than the 20% rate in the Dutch population aged 55 years and older.^57^ Therefore, the true incidence of morbidity in the older traveller might be higher. For that reason, we also compared the data of our group travellers aged between 70 and 79 years (*n* = 117) with the data from individuals of the same age group of the AT-AGE study (*n* = 303) in which an identical measurement procedure had been used in different primary care practices.^58^ Linear regression demonstrated no significant mean difference in the maximal grip strength between both cohorts, after correcting for age and gender (mean difference 1.3, 95% CI −0.2 to 2.8, *P* = 0.096). This implied that the older travellers visiting the clinic pre-travel physically did not differ (much) from the older adults visiting primary care practices. Of interest, a recent retrospective study among older travellers visiting an Irish travel health clinic pre-travel demonstrated similar health (e.g. about one third used no medication, majority had a medical condition) and travel characteristics (e.g. South Eastern Asia and South America as popular destinations, travel duration of 3 weeks, mostly traveling for leisure or visiting friends or family) as in this ELDEST cohort.^59^

### In practice

This study demonstrates that older Dutch travellers to the tropics are generally fit, well-prepared and experienced relatively low rates of morbidity. Although several travellers encountered travel-related morbidity, these travellers did not solely entail illness, as the participants perceived both improved mental- and physical health after travel. Special attention should be given to travellers with the identified predictors (e.g. long travel duration, destinations in Northern Africa or South-East and East Asia, high CCI score, multiple daily medications, using a mobile phone and media use). Furthermore, extensive travel experience should not reassure the travel advisor.

## Supplementary Material

Supplement_manuscript_ELDEST_Vlot_taaa216

## Abbreviations

DM (Diabetes Mellitus), ELDEST (morbidity in ELDErly travellers during a Short-Term stay abroad), LUMC (Leiden University Medical Centre), MHSs (Municipal Health Services), STROBE (Strengthening the Reporting of Observational studies in Epidemiology), 6CIT (Six item Cognitive Impairment Test), SF-36 (Short Form 36 health survey), Katz-ADL (Katz Activities of Daily Living), CCI (Charlson Comorbidity Index), WHO (World Health Organization), SPSS (Statistical Package for the Social Sciences), IQR (interquartile range), GP (General Practitioner), RTI (Respiratory Tract Infection), COPD (Chronic Obstructive Pulmonary Disease), GE (Gastroenteritis), RR (Relative Risk), CI (confidence interval), ORS (Oral Rehydration Solution), AT-AGE (Age and Thrombosis: Acquired and Genetic risk factors in the Elderly).

## Notes

Poster presentation with preliminary data of this study at the 15th Conference of the International Society of Travel Medicine, 14–18 May 2017; Barcelona, Spain.Oral presentation at the 16th Conference of the International Society of Travel medicine, 5–9 June 2019; Washington DC, the United States of America.

Poster presentation with preliminary data of this study at the 15th Conference of the International Society of Travel Medicine, 14–18 May 2017; Barcelona, Spain.

Oral presentation at the 16th Conference of the International Society of Travel medicine, 5–9 June 2019; Washington DC, the United States of America.
