# Supplementary material for: Predicting morbidity in older travellers during a short-term stay in the tropics: the ELDEST study
Source: J Travel Med. 2020 Nov 19;28(1):taaa216. doi: 10.1093/jtm/taaa216 (PMC7788562; doi:10.1093/jtm/taaa216)
Supplement: Supplement_manuscript_ELDEST_Vlot_taaa216 [file supplement_manuscript_eldest_vlot_taaa216.docx]

**Supplement**

**Predicting morbidity in older travelers during a short-term stay in the tropics: the ELDEST study**

*Running title: Predictors of morbidity in older travelers during a tropical journey*

Jessica A. Vlot, MSc^1^; Marissa G.D. Vive, MSc^1^; Henricus J. Brockhoff, MD^2^; Pieter J.J. van Genderen, MD, PhD^3;^ Marie-Christine E. Trompenaars, MD^4^; James E. van Steenbergen, MD, PhD^1^; and Leonardus G. Visser, MD, PhD^1^.

^1^ Department of Infectious Diseases, Leiden University Medical Center, Leiden, the Netherlands.

^2^ Department of Infectious Diseases, Municipal Health Service, The Hague, the Netherlands.

^3^ Harbour Hospital and Institute for Tropical Diseases, currently working on Department of Medical Microbiology and Infectious Diseases, Erasmus MC, Rotterdam Netherlands.

^4^ Department of Infectious Diseases, Municipal Health Service, Rotterdam, the Netherlands.

**Table and Figure contents**

**eTable** **1**. Demographic and travel characteristics of a cohort of 477 older travelers to the tropics.

**eTable** **2**. Travel preparation of a cohort of 477 older travelers to the tropics

**eTable** **3**. Risk behavior in a cohort of 477 older travelers to the tropics

**eTable** **4**. Malaria prophylaxis in a cohort of 477 elderly traveling to the tropics

**eTable 5**. Morbidity documented in questionnaires during and up to two weeks after returning home in 477 older travelers to the tropics.

**eTable 6**. Changes in self-perceived health after vs pre-travel using SF-36 in 477 older travelers to the tropics

**eTable 7**. Comparison of available and partly available cases of 649 older travelers to the tropics

**eFigure 1**. Most visited destinations of 477 older Dutch travelers (≥60 years) traveling to the tropics for a short-term stay.

**eAppendix 1**. Protocol functional tests measurements

**eAppendix 2**. Standardized tests to identify potential risk profiles

**eAppendix 3**. Symptom clusters for health complaints in diaries

**eTable 1.** Demographic and travel characteristics of a cohort of 477 older travelers to the tropics.

|  | **Available cases ^a^**  **N=477** |  |
| --- | --- | --- |
| **Age,** *years, median (IQR)* | 66 (63-70) |  |
| **Age group** |  |  |
| 60-69 y | 345 (72) |  |
| 70-79 y | 117 (25) |  |
| ≥80 y | 15 (3) |  |
| **Gender,** *female* | 232 (49) |  |
| 60-69y | 174/345 (50) |  |
| 70-79y | 53/117 (45) |  |
| ≥80y | 5/15 (33) |  |
| **Education level**^b^ |  |  |
| Primary education | 38 (8) |  |
| Secondary education | 162 (34) |  |
| Higher education | 277 (58) |  |
| **Immigrant** | 39 (8) |  |
| **Travel experience to tropical destinations**^c^ | 437 (92) |  |
| If travel experience, number of journeys in preceding 5 years |  |  |
| 0 | 99 (21) |  |
| 1-5 | 280 (59) |  |
| 6-10 | 28 (6) |  |
| >10 | 28 (6) |  |
| **Most visited tropical destinations**^de^ |  |  |
| South-Eastern Asia | 163 (34) |  |
| Southern Asia | 68 (14) |  |
| South America | 58 (12) |  |
| Eastern Africa | 51 (11) |  |
| Southern Africa | 45 (9) |  |
| Western Africa | 31 (6) |  |
| Central America | 22 (5) |  |
| **Travel duration,** days, *median (IQR)* | 19 (14-23) |  |
| <1 week | 6 (1) |  |
| 1-2 weeks | 117 (25) |  |
| 2-3 weeks | 181 (38) |  |
| 3-4 weeks | 113 (24) |  |
| 4-5 weeks | 60 (13) |  |
| **Purpose of travel** |  |  |
| Holiday | 380 (80) |  |
| Visiting friends or relatives | 70 (15) |  |
| Business | 15 (3) |  |
| Volunteering | 12 (3) |  |
| **Most frequent travel group composition** |  |  |
| With partner | 237 (50) |  |
| With an organized group travel | 96 (20) |  |
| With friends | 36 (8) |  |
|  |  |  |
|  | **Available cases ^a^**  **N=477** |  |
| Solo | 30 (6) |  |
| With children | 23 (5) |  |
| **Most frequent type of accommodation during travel**^e^ |  |  |
| Hotel | 346 (73) |  |
| Lodge | 66 (14) |  |
| With friends/family | 48 (10) |  |
| Guesthouse | 29 (6) |  |
| Cruiseship | 18 (4) |  |
| **Used a mobile phone** | 466 (98) |  |
| Smartphone^c^ | 397 (85) |  |
| **Social media use**^ce^ | 285 (60) |  |
| Most frequently used |  |  |
| Facebook | 221 (47) |  |
| LinkedIn | 136 (29) |  |
| YouTube | 51 (11) |  |

Data represent absolute numbers (no.) and percentages per category (%), unless otherwise specified.

^a^ All eligible travelers that participated in part B and were not lost to follow-up before questionnaire 2 were designated available cases, while all other eligible travelers were designated unavailable cases.

^b^ Education level: according to classification Central Bureau for Statistics, the Netherlands.

^c^ Percentages were calculated over the total number of travelers that answered the concerning question. Some travelers did not fill in the questions concerning these items, resulting in a maximum of 3 missing values.

^d^ Travel destination: categorized according to geographical regions described by the United Nations Statistics Division.

^e^ Totals may exceed 100% since multiple options per participant can apply

| **eTable 2.** Travel preparation of a cohort of 477 older travelers to the tropics. | | |
| --- | --- | --- |
|  | **Available cases** |  |
|  | **N=477** |  |
| **Method of booking** |  |  |
| Online | 287 (60) |  |
| Travel agency | 165 (35) |  |
| Telephone | 17 (4) |  |
| **Duration between pre-travel consult and departure,** *days, median (IQR)* | 37 (21-51) |  |
| **Most frequent additional pre-travel advice sought ^a^** |  |  |
| General practitioner | 432 (91) |  |
| Internet | 84 (18) |  |
| Travel bureau | 38 (8) |  |
| Pharmacy | 36 (8) |  |
| Medical specialist | 17 (4) |  |
| **Insurance status** |  |  |
| Travel insurance | 464 (97) |  |
| Including medical costs | 413 (87) |  |
| Including repatriation | 417 (89) |  |
| Health insurance covers medical costs abroad | 416 (89) |  |
| **Medicines and travel** (n=318) **^b^** |  |  |
| Took extra supply of regular medication | 257 (81) |  |
| Travelled with personal prescription information sheet | 248 (78) |  |
| **Medication against GI complaints in carry-on bag^a^** |  |  |
| Loperamide | 347 (73) |  |
| ORS | 284 (60) |  |
| Activated carbon | 89 (19) |  |
| Antibiotics | 62 (13) |  |
| **Hand hygiene products in carry-on bag^a^** |  |  |
| Hand sanitizer | 275 (58) |  |
| Wet wipes | 187 (39) |  |
| Soap | 127 (27) |  |
| Data represent absolute numbers (no.) and percentages (%), unless otherwise specified. GI, gastrointestinal complaints. Percentages were calculated over the total number of travelers that answered the concerning question. Some travelers did not fill in the questions concerning these items, resulting in a maximum of 10 missing values. Abbreviations: ORS, Oral Rehydration Solution.  ^a^ Total may exceed 100% since multiple options per participant can apply.  ^b^ Within group of travelers that used medication. | | |

**eTable 3.** Risk behavior in a cohort of 477 older travelers to the tropics.

|  | **Available cases** | |
| --- | --- | --- |
|  | **N=477** | |
| **Washing hands regularly ^a^** |  |  |
| After using the toilet | 459 | (96) |
| Before meal | 400 | (84) |
| **Using hand sanitizer regularly^◊^** | 207 | (43) |
| **Using tap water for toothbrushing** | 251 | (53) |
| **Using bottled water from sealed bottle for toothbrushing** | 254 | (53) |
| **Hand contact with animals (e.g. stroking or holding)^a^** | 97 | (20) |
| Monkey | 88 | (91) |
| Dog | 47 | (48) |
| Cat | 28 | (29) |
| Elephant | 14 | (14) |
| Snake | 6 | (6) |
| Camel/dromedary | 5 | (5) |
| **Drinking/eating ^a^** |  |  |
| Food from hotel buffay | 381 | (80) |
| Fruit that was not cleaned by participant | 362 | (76) |
| Freshly squeezed juices | 311 | (65) |
| Salad prepared by others | 302 | (63) |
| Drinks with icecubes | 216 | (45) |
| Soft ice | 126 | (26) |
| Food from food stands | 92 | (19) |
| Raw crustaceans | 43 | (9) |
| Products containing raw eggs | 37 | (8) |
| Leftovers from previous meal | 32 | (7) |
| Raw shellfish | 27 | (6) |
| Non pasteurized cheese | 21 | (4) |
| Raw or medium rare meat | 18 | (4) |
| Pasteurized milk | 7 | (1) |
| **Barefoot walking ^a^** |  |  |
| Only at the beach | 204 | (43) |
| On the street | 34 | (7) |
| **Swimming in the sea** | 146 | (31) |
| **Had contact with mud or soil** | 165 | (35) |
| **Visited a local market** | 369 | (78) |

Data represent absolute numbers (no.) and percentages (%). Percentages were calculated over the total number of travelers that answered the concerning question. Some travelers did not fill in the questions concerning these items, resulting in a maximum of four missing values. Percentages were calculated over the total number of travelers that answered the question.

^a^ Total may exceed 100% since multiple options per participant can apply.

**^◊^** 20 travelers used a hand sanitizer they did not brought themselves.

**eTable 4.** Malaria prophylaxis in a cohort of 477 older travelers to the tropics.

|  | **Available cases** |
| --- | --- |
|  | **N=477** |
| **Malaria chemoprophylaxis started** |  |
| No, it was not recommended by the clinic | 330 (69) |
| No, but it was recommended by the clinic^*^ | 27 (6) |
| Yes | 120 (25) |
| **Type of chemoprophylaxis** |  |
| Atovaquone/proguanil | 112 (93) |
| Mefloquine | 1 (1) |
| Proguanil | 7 (6) |
| **Side effects** |  |
| Atovaquone/proguanil | 18 (15) |
| Mefloquine | 0 |
| Proguanil | 0 |
| **Type of side effects from atovaquone/proguanil** ^a^ |  |
| Gastrointestinal complaints | 9 (50) |
| Abdominal pain | 3 |
| Nausea | 3 |
| Diarrhoea | 3 |
| Sleeping complaints | 7 (39) |
| Sleeplessness | 3 |
| Strange/vivid dreams | 4 |
| Dizziness | 6 (33) |
| Headache | 1 (6) |
| **Compliance malaria chemoprophylaxis** |  |
| Full compliance | 110 (92) |
| **Reasons for non compliance** |  |
| Side effects | 4 (30) |
| Too few tablets brought | 2 (20) |
| Other^**^ | 4 (40) |
| **Bed net used ^b ***^** |  |
| No, it was not recommended by the clinic | 329 (69) |
| No, but it was recommended by the clinic | 29 (6) |
| Yes | 117 (25) |

Data represent absolute numbers (no.) and percentages (%).

^a^ Total may exceed 100% since patients could report multiple side effects.

^b^ Percentages were calculated over the total number of travelers that answered the concerning question. Some travelers did not fill in the questions concerning these items, resulting in a maximum of two missing values.

^*^One traveler got robbed of personal belongings, including the chemoprophylaxis. One traveler brought the chemoprophylaxis as standby emergency treatment. Another traveler did so due to low temperatures and no mosquitos were noticed.

^**^Only in specific areas (n=2), staying on altitude (n=1), unknown reason (n=1).

^***^Main reason for not using a bed net was the presence of a functioning air-conditioning.

**eTable 5.** Morbidity documented in questionnaires during and up to two weeks after returning home in 477 older travelers to the tropics.

|  | **Available cases**  **N=477** |
| --- | --- |
| **Injury** | 61 (13) |
| Fallen | 18 |
| Cut | 10 |
| Bitten, licked or scratched by animal | 1 |
| Other minor injuries | 32 |
| **Exacerbation of chronic health complaints** | 22 (5) |
| **Travelers’ diarrhea (subjective)**^a^ | 141 (30) |
| Medication temporariliy discontinued | 10 (2) |
| Diuretics (n=19) | 3 |
| Antihypertensives (n=49) | 2 |
| Statins (n=40) | 5 |
| **Obstipation^b^** | 34 (7) |
| **Skin complaints ^b^** | 59 (12) |
| Exacerbation of chronic skin disease | 10 (17) |

Data represent absolute numbers (no.) and percentages (%).

^a^ Travelers’ diarrhea was defined as the passage of three or more unformed stools during a 24-hour period.

^b^ Percentages were calculated over the total number of travelers that answered the concerning question. Some travelers did not fill in the questions concerning these items, resulting in a maximum of two missing values.

**eTable 6.** Changes in self-perceived health after vs pre-travel versus using SF-36 in 477 older travelers to the tropics.

|  | **Post-travel** (median, IQR) | **Pre-travel** (median, IQR) | **p value ^b^** | **Individual difference^c^** (median, IQR) | **Interpretation** Change in self-perceived health (S/NS) |
| --- | --- | --- | --- | --- | --- |
| **Mental health sum score** | **91 (86; 95)** | **89 (83; 93)** | **0.00** | **+3 (-2; 6)** | **Improved mental health** (S) |
| Vitality (energy/fatigue) | 85 (75; 90) | 80 (70; 90) | 0.00 | +5 (-5; 10) | Improved vitality (S) |
| Social functioning | 100 (100; 100) | 100 (100; 100) | 0.15 | +13 (-13; 13) | Improved social functioning (NS) |
| Role emotional | 100 (100; 100) | 100 (100; 100) | 0.86 | -33 (-33; 33) | More impairment due to emotional problems (NS) |
| General mental health ^a^ | 92 (84; 96) | 88 (80; 92) | 0.00 | +4 (-4;8) | Improved mental health (S) |
| **Physical health sum score** | **91 (82; 95)** | **89 (82; 94)** | **0.01** | **+1 (-4; 5)** | **Improved physical health** (S) |
| Physical functioning | 95 (85; 100) | 95 (85; 100) | 0.23 | +5 (-5; 5) | Improved physical functioning (NS) |
| Role physical | 100 (100; 100) | 100 (100; 100) | 0.83 | +25 (-50; 50) | Less impairment due to physical problems (NS) |
| Bodily pain | 100 (87; 100) | 88 (75; 100) | 0.00 | +13 (13; 25) | Less bodily pain (S) |
| General health  perceptions | 75 (65; 85) | 75 (65; 85) | 0.58 | +5 (-10; 10) | Improved general health (NS) |

Data represent median SF-36 scores (range 0-100). Abbreviations: SF-36, Short-Form 36 survey; S, significant; NS, not significant.

^a^ One participant did not answer the question concerning this item, resulting in one missing value.

^b^ Analysis of differences between post and pre-travel SF-36 scores was performed by Wilcoxon signed rank test.

^c^ Positive values correspond with an health improvement within travelers, while negative values correspond with a decrease in health.

**eTable 7.** Comparison of available and partly available cases of 649 older travelers to the tropics.

|  | **Available cases**^a^ | | **Partly available cases**^a^ | | **Comparison**^b^ |  |
| --- | --- | --- | --- | --- | --- | --- |
|  | **N = 477** | | **N = 172** | | **p value** |  |
| **Gender,** female | 232 | (49) | 87 | (51) | 0.72 |  |
| **Age,** median, *years (IQR)* | 66 | (63-70) | 67 | (63-71) | 0.06 |  |
| **Age groups** |  |  |  |  | **0.19** |  |
| 60-69 y | 345 | (72) | 112 | (65) |  |  |
| 70-79 y | 117 | (25) | 52 | (30) |  |  |
| ≥80 y | 15 | (3) | 8 | (5) |  |  |
| **Grip strength,** *kg, median (IQR)^c^* | 34 | (28-45) | 33 | (26-42) | 0.046 |  |
| **6CIT total score,** *median (IQR)^d^* | 0 | (0-2) | 2 | (0-4) | 0.001 |  |
| **Most visited tropical destinations**^ef^ |  |  |  |  | 0.03 |  |
| South-Eastern Asia | 163 | (34) | 40 | (23) |  |  |
| South America | 58 | (12) | 36 | (21) |  |  |
| Southern Asia | 68 | (14) | 22 | (13) |  |  |
| Eastern Africa | 51 | (11) | 15 | (9) |  |  |
| Southern Africa | 45 | (9) | 20 | (12) |  |  |
| Western Africa | 31 | (6) | 7 | (4) |  |  |
| Central America | 22 | (5) | 3 | (2) |  |  |
| Western Asia and Middle East | 14 | (3) | 7 | (4) |  |  |
| Northern Africa | 13 | (3) | 7 | (4) |  |  |
| Eastern Asia | 14 | (3) | 4 | (2) |  |  |
| Carribean | 12 | (3) | 6 | (3) |  |  |
| Central and Middle Africa | 10 | (2) | 1 | (1) |  |  |
| Central Asia | 5 | (1) | 0 | (0) |  |  |
| Eastern Europe | 1 | (0) | 1 | (1) |  |  |
| Data represent absolute numbers (n) and percentages per category (%), unless otherwise specified.  Abbreviations: 6CIT, Six Item Cognitive Impairment Test.  ^a^ All eligible travelers that participated in part A only or were lost to follow-up before questionnaire 2 were designated partly available cases, while all other eligible travelers were designated available cases.  ^b^ Mann Whitney U test was used for analysis of continuous variables. Pearson's Chi-square test was used for analysis of categorical variables.  ^c^ Grip strength: Range 0-90 kg, see eAppendix 1.  ^d^ 6CIT: Range 0-28, see eAppendix 1.  ^e^ Travel destination: categorized according to geographical regions described by the United Nations Statistics Division.  ^f^ Totals may exceed 100% since some travelers visited multiple destinations. | | | | | | |

**eFigure 1.** Most visited destinations of 477 older Dutch travelers (≥60 years) traveling to the tropics for a short-term stay**.**

**
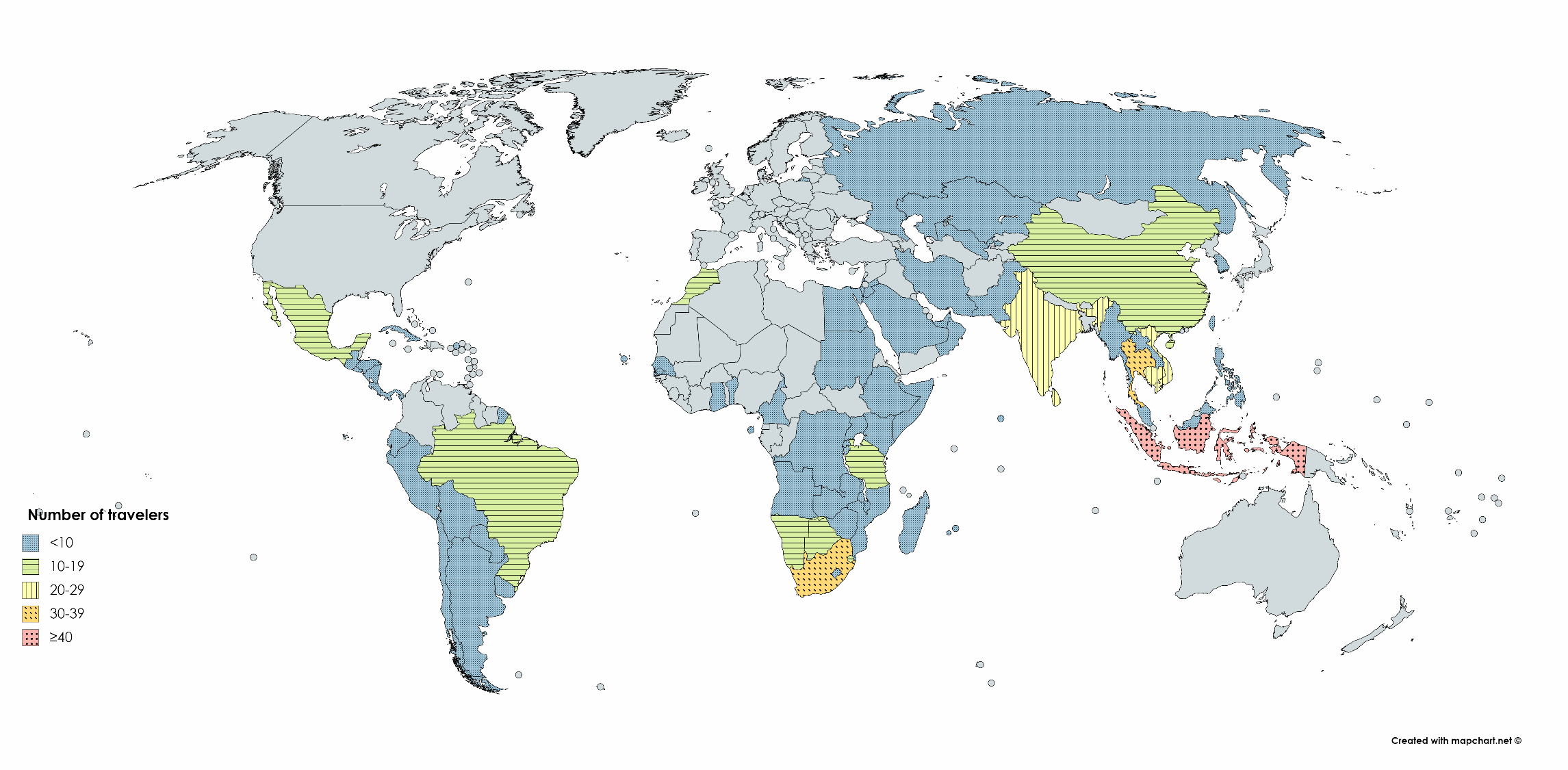
**

**eAppendix 1.** Protocol functional tests measurements

Hand grip strength measurement protocol

| 1. | Participant sit in a chair with back support and without arm rests. |
| --- | --- |
| 2. | The arm/elbow of the participant should be at 90˚, thumbs facing upwards (Figure 1) |
| 3. | Measurement is taken by the dominant hand (participant is asked ‘which hand do you use to write’). If the participant is not able to use the dominant hand, the non-dominant hand is used. The reason for not using the dominant hand is noted. For all measurements, the dynamometer was set to the second handle position from the inside, as most people squeeze on their maximum power in this position.^1^ |
| 4. | The participant is encouraged to squeeze the Jamar hand dynamometer (Figure 2) as tightly as possible for 2-3 seconds. |
| 5. | Grip strength in kilograms is read from the outside dial and recorded on the data entry form (range of 0-90 kg). |
| 6. | In total four measurements are performed of which the first one is a test measurement and is not noted on the form. |
| 7. | In order to interfere as little as possible with regular practice in the travel clinics, hand grip strength was measured after travel consultation and administration of vaccinations. |

|  |  |
| --- | --- |
| Figure 1 | Figure 2 |

Six Item Cognitive Impairment Test (6CIT, range 0-28 points)

| **Question** | **Score range** | **Score** |  |
| --- | --- | --- | --- |
| 1. What year is it? | Correct – 0 points  Incorrect – 4 points |  |  |
| 1. What month is it? | Correct – 0 points  Incorrect – 3 points |  |  |
| *Give the patient an address phrase to remember*  *e.g. Jan, de Vries, Molenstraat 12, Groningen* | | | |
| 1. What time is it (without looking at the clock)   *A margin of 1 hour* | Correct – 0 points  Incorrect – 3 points |  |  |
| 1. Count backwards from 20 to 1 | Correct – 0 points  1 error – 2 points  >1 error – 4 points |  |  |
| 1. Say the months of the year in reverse   *Dec, Nov, Oct, Sep, Aug, Jul, Jun, May, Apr, Mar, Feb, Jan* | Correct – 0 points  1 error – 2 points  >1 error – 4 points |  |  |
| 1. Repeat address phrase   *Score:* *[Jan] [de Vries] [Molenstraat] [12] [Groningen]* | Correct – 0 points  1 error – 2 points  2 errors – 4 points  3 errors – 6 points  4 errors – 8 points  All wrong – 10 points |  |  |
| **Total score** | **0 - 28** | **___ / 28** |  |

**eAppendix 2.** Standardized tests to identify potential risk profiles

**SF-36 health survey including eight health concepts^2^:**

*Physical health sum score*

- Physical functioning;
- Role limitations because of physical health problems;
- Bodily pain; and
- General health perceptions.

*Mental health sum score*

- Vitality (energy/fatigue);
- Social functioning;
- Role limitations because of emotional problems
- General mental health;

**Katz-ADL^3,4^**

| **Question** | **Answers (score)** |
| --- | --- |
| Receiving assistance in bathing? | yes (1) / no (0) |
| Receiving assistance getting dressed? | yes (1) / no (0) |
| Receiving assistance in going to the toilet? | yes (1) / no (0) |
| Using incontinence pads? | yes (1) / no (0) |
| Receiving assistance moving from bed to chair? | yes (1) / no (0) |
| Receiving assistance in feeding? | yes (1) / no (0) |
|  |  |

**Charlson Comorbidity Index (CCI),** a tool to measure comorbidity which has been used previously to estimate 10-year survival.^5,6^

| **Item** | **CCI Score** |
| --- | --- |
| Myocardial infarction | 1 |
| Congestive heart failure | 1 |
| Peripheral vascular disease | 1 |
| Cerebrovascular disease | 1 |
| Dementia | 1 |
| Chronic pulmonary disease | 1 |
| Connective tissue disease | 1 |
| Ulcer disease | 1 |
| Mild liver disease | 1 |
| Diabetes mellitus | 1 |
| Hemiplegia | 2 |
| Moderate or severe renal disease | 2 |
| Diabetes mellitus with end organ damage | 2 |
| Any tumor | 2 |
| Leukemia | 2 |
| Lymphoma | 2 |
| Moderate or severe liver disease | 3 |
| Metastatic solid tumor | 6 |
| Acquired immunodeficiency syndrome | 6 |

**eAppendix 3.** Symptom clusters for health complaints in diaries.^7^

| **Symptom cluster** | **Criteria** | **Restriction** | **Duration** |
| --- | --- | --- | --- |
| General infections | Fever (temperature ≥ 38.0°C). | Not fulfilling criteria of another symptom cluster |  |
| Uncomplicated gastroenteritis | Watery or unformed stools more than three times per 24 hours and one or more of the following complaints; nausea, vomiting, abdominal cramps and fecal incontinence. | No fever or bloody stools. | Maximum: 14 days |
| Complicated gastroenteritis | Watery or unformed stools more than three times per 24 hours and one or more of the following complaints; fever and bloody stools.  Or  Uncomplicated gastroenteritis that lasted longer than 14 days. |  |  |
| Dehydration | Two or more of the following; urination less than four times per 24 hours, dark urine, thirst, dry mucosae and orthostatic dizziness. |  |  |
| Uncomplicated respiratory tract infection | Cough and one or more of the following complaints: nasal constipation, sore throat and coughing up sputum. | No dyspnea, pain on the chest or heart palpitations | Maximum: 7 days |
| Complicated respiratory tract infection | Cough and fever and one or more of the following complaints; dyspnea at rest or pain on the chest related to respiration  Or  Uncomplicated respiratory tract infection that lasted longer than 7 days | No heart palpitations and the pain on the chest was not elicited by exercise |  |
| Uncomplicated urinary tract infection | Painful urination | No fever | Maximum: 7 days |
| Complicated urinary tract infection | Two or more of the following; painful urination, fever and flank pain  Or  Uncomplicated urinary tract infection that lasted longer than 7 days |  |  |
| Angina pectoris | Pain on the chest elicited by exercise | No fever or pain on the chest related to respiration |  |
| Cardiac failure | Swollen ankles or lower legs and dyspnea at rest |  |  |
| Peripheral edema | Swollen ankles or lower legs | No dyspnea | Minimum: 3 days |
| Musculoskeletal complaints | Knee-, neck/shoulder- or back pain |  |  |

**References**

1. Trampisch US, Franke J, Jedamzik N, Hinrchs T, Platen P. Optimal Jamar dynamometer handle position to assess maximal isometric hand grip strength in epidemiological studies. J Hand Surg Am. 2012 Nov;37(11):2368-73.
2. Meetinstrumenten zorg. RAND-36 item Health Survey, Medical Outcomes Study 36-Item Short Form Health Survey. <http://www.meetinstrumentenzorg.nl/algemenemeetinstrumenten.aspx?meetinstrument=47>
3. Katz S, Ford AB, Moskowitz RW, Jackson BA, Jaffe MW. Studies of Illness in the Aged. The Index of Adl: A Standardized Measure of Biological and Psychosocial Function. *JAMA.* 1963;185:914-919.
4. Katz-ADL6. Available online at http://www.vmszorg.nl/_library/5512/screeningsinstrument%20Katz- ADL6%20(Katz%201963)%2023-05-2011.pdf
5. Charlson ME, Pompei P, Ales KL, MacKenzie CR. A new method of classifying prognostic comorbidity in longitudinal studies: development and validation. *J Chronic Dis.* 1987;40(5):373-383.
6. Frenkel WJ, Jongerius EJ, Mandjes-van Uitert MJ, van Munster BC, de Rooij SE. Validation of the Charlson Comorbidity Index in acutely hospitalized elderly adults: a prospective cohort study. *J Am Geriatr Soc.* 2014;62(2):342-346. doi:10.1111/jgs.12635.
7. The Dutch College of General Practitioners. Nhg-standaarden. https://www.nhg.org/nhg-standaarden (October 2018, date last accessed, In Dutch).
